# “It Seems Much More Enjoyable Now”: Parental Perception of Relational Change from Participating in Paediatric Autism Communication Therapy (PACT)

**DOI:** 10.3390/children11070838

**Published:** 2024-07-10

**Authors:** Charlotte Engberg Conrad, Rikke Jørgensen, Cecilie Amstrup, Tine Ellitsgaard Gottschau, Per Hove Thomsen, Marlene Briciet Lauritsen

**Affiliations:** 1 Psychiatry, Aalborg University Hospital, 9000 Aalborg, Denmark; rijo@rn.dk (R.J.); cecilie.amstrup@rn.dk (C.A.); marlene.lauritsen@rn.dk (M.B.L.); 2 Department of Clinical Medicine, Aalborg University, 9260 Gistrup, Denmark; 3 Research Unit, Department of Child and Adolescent Psychiatry, Copenhagen University Hospital—Psychiatry Region Zealand, 4000 Roskilde, Denmark; tgot@regionsjaelland.dk; 4 Department of Child and Adolescent Psychiatry, Aarhus University Hospital, Psychiatry, 8200 Aarhus, Denmark; per.hove.thomsen@psykiatrien.rm.dk; 5 Department of Clinical Medicine, Aarhus University, 8000 Aarhus, Denmark

**Keywords:** autism, autism spectrum disorders, neurodevelopmental disorders, parent-mediated intervention, Paediatric Autism Communication Therapy, PACT, qualitative study, semi-structured interviews

## Abstract

Objectives: This qualitative study aims to examine parental experiences of feasibility and relational changes from participating in the Paediatric Autism Communication Therapy (PACT) intervention. Methods: Thirteen parents of children diagnosed with autism spectrum disorder (mean age 3.89 years) participated in semi-structured interviews. Thematic analysis was performed, inspired by an abductive approach informed by the theories of the attachment system, the caregiving system and mentalisation. Results: Three overarching themes were identified: the struggle of fitting PACT into everyday life, the fruit of relational connection and the cascading relational effects of PACT. Parents were challenged regarding finding time for the intervention but adapted PACT to their individual needs and possibilities. All parents experienced relational improvement, and a cycle of positive relational change through PACT was identified. Conclusions: This study has several clinical implications. Therapists and clinics offering PACT interventions should encourage and support parents in their individual journey of implementing PACT into their everyday lives. Some of the parents described improvements in parental mentalisation, child attachment and mutual enjoyment in the parent–child relationship. Children with autism could benefit from parents increasing their sensitivity when caregiving, and clinicians may through interventions such as PACT facilitate this development.

## 1. Introduction

Autism is a heterogeneous neurodevelopmental disorder describing individuals who have a combination of disabilities in social interaction, communication and repetitive restricted behaviours [1]. Autism affects 1% of the population worldwide and has a higher prevalence in Western countries [1,2]. Interventions supporting social communication development—especially parent-mediated interventions such as Paediatric Autism Communication Therapy (PACT) aiming at improving dyadic communication between a caregiver and child—are imperative, as they are cost-effective and offer the opportunity to generalise skills and learning opportunities into everyday life [3,4].

PACT is a parent-mediated video-aided intervention aimed at improving language and social communication in children with autism [3,5]. Theoretically, PACT is rooted in developmental theory [6], and previous research has shown that the PACT intervention may reduce autism symptomatology as measured by the Autism Diagnostic Observation Schedule (ADOS) in children aged 2–4 years diagnosed with core autism 13 months from baseline [3]. A follow-up study after 6 years from baseline has demonstrated that the change of reduced autism symptom severity was both sustained over time and that the developmental trajectory of the PACT treatment group over time looked improved compared to the control group [7]. Sustained change in the autistic child’s initiation of social interaction was the main mediator of reduction in autism symptomatology [8]. In a study of Paediatric Autism Communication Therapy-Generalised (PACT-G) in the UK, PACT was further investigated in children aged 2–10 years and mediated through therapists, parents and learning support assistants at school [9]. The results of this particular study did not replicate those of the original study on the primary outcome of autism symptom severity, and the effects on dyadic communication were less strong, possibly due to the increased age of the children as well as the change in the number of sessions with parent and therapist participation [3,9,10]. Nonetheless, significant improvement in parental well-being and reduction in child disruptive behaviour was also found [9]. As PACT improves social communication, it is likely that relational aspects in the family could be positively affected as well.

Attachment is a key relational construct. Attachment theory was developed by John Bowlby and is a theory of the innate biological human capacity to form lasting relational bonds. The attachment system is activated when the child seeks comfort, proximity and security to the primary caregiver [11,12]. Two studies have examined whether an intervention could hold the capacity to change the attachment system of the child when the caregiver is aided in demonstrating more parental sensitivity when communicating with the child with autism [13,14]. A study of two cases of parent–child dyads participating in the attachment-based intervention Circle of Security showed that one of the children changed attachment category from dismissing to secure, while the other participant remained secure [13]. In a randomised controlled trial (RCT) of a developmental intervention, the Focused Playtime Intervention demonstrated a dimensionally higher level of security in the intervention group compared to management as usual [14].

In 2008, attachment researchers George and Solomon elaborated the framework of the caregiving system as a behavioural system of the caregiver providing care for the child [15]. The caregiving system describes an organised set of behaviours informed by the parent–child relationship and a complex transaction between experiential and biological factors [15]. The characteristics of the child with autism most likely affect the caregiving system, and the experiential changes that PACT facilitates may impact this.

Reflective functioning—the ability to understand mental states of the self and others—evolves in the interaction with a caregiver who has the ability to mentalise, understand and regulate the inner world of the child. Reflective function is considered one of the mediators of attachment representation transmission from one generation to the next when parents simultaneously can reflect on the subjective state of the child and act accordingly [16]. Parental mentalising refers to the parents’ capacity to understand the internal mental states of their children and reflect upon how their own inner world is shaped and changed by interaction with their child [17]. Social–communicative disabilities of a child with autism may make it more difficult for the parent to comprehend mental states and initiatives taken by the child, while the behaviour of parents will be more difficult for the child to predict and understand [18,19]. To date, whether PACT affects parental mentalising, attachment or the caregiving system has not been investigated. However, since PACT directly aims to affect the child through increased parental sensitivity, a positive change in attachment security, mentalising capacity and caregiving seems likely.

In 2020, Leadbitter et al. performed a qualitative phenomenological study of PACT [20] that involved the participation of parents from the RCT [3]. The study showed perceived positive relational change in parents’ interaction and relationship with their child. Parents additionally experienced improvements in their child’s communication [20]. Leadbitter et al. [20] emphasised important aspects not examined in their study, including the role of the non-participating parent in implementing PACT strategies in daily living with the child, experience of the daily training at home and goodness of fit of the PACT intervention with other approaches on offer in community or educational settings. The present study will accommodate some of these research gaps in addition to further elaborating on the relational aspects of participating in PACT for the children with autism and their families who participated in a feasibility study of the PACT intervention in Denmark.

The aim of this study was twofold:

1. To examine the parental perception of relational changes through the PACT intervention. This could be subjective experiences of change in the child’s attachment behaviour, parental mentalising capacity, caregiving or any lived experience of change in the relationship both between the participating parent and child dyad. This was also examined with attention being given to other relationships in the child’s daily life.
2. To describe the parental experience of the feasibility of the PACT intervention and potential negative effects.

## 2. Materials and Methods

This study is a qualitative interview study rooted in the methodology of pragmatism [21].

### 2.1. Setting

A feasibility study was performed in all five regions of Denmark prior to a large randomised clinical Danish trial of PACT. This qualitative interview study was conducted as part of the feasibility study. Participants for the feasibility study were recruited at the time the child received a diagnosis of autism spectrum disorder at a child psychiatric department in any of the five regions of Denmark. Inclusion criteria for participating in the study were that the children were diagnosed with autism spectrum disorder according to ICD-10 with a score of 4 or more on the Autism Diagnostic Observation Schedule calibrated severity score (ADOS CSS), children not having a severe hearing disability and parents being able to communicate in Danish or English. In the feasibility study, the families were asked to choose one parent to be the primary participant in the intervention. The primary parent agreed to participate in all PACT sessions with the therapist and to be videotaped interacting with their child. Additionally, this parent agreed to participate in baseline and endpoint assessments with their child.

### 2.2. The Intervention

PACT is a parent-mediated intervention with the parent changing their behaviour to make the social–communicative environment more supportive and well-suited to the child’s communication, which in turn improves the child’s social communication. The intervention consists of 12 sessions every two weeks for six months and is followed by six monthly sessions [3,7]. During the feasibility study, the first 12 sessions were mandatory, while the remaining six booster sessions were optional. Parents were additionally asked to practice the principles at home for 30 min daily, videotape their interaction with their child and bring the recording to the therapy sessions. During the sessions, therapists would use positive attribution, i.e., where the therapist emphasized elements of synchronous parent communication and mutual shared attention between parent and child. The intervention could be delivered online or in person. The PACT therapist introduced the parent to the 6 developmental steps of communication:

1. Establishing shared attention
2. Synchrony and sensitivity
3. Focusing on language input
4. Establishing routines and anticipation
5. Increasing communication functions
6. Language expansion and developing conversation

PACT is individualised to the specific child’s development and the parent’s preferred learning style. In the feasibility study, parents recorded interaction with their child at home and worked in partnership with their therapist using both parents’ knowledge of their child and the therapist’s professional knowledge [3,7]. In our sample, none of the children participated during the sessions. Five of the participating families received the PACT intervention online, while four families had direct contact with their therapist. One family used a combination of online and direct contact with a therapist. All participants in this study received a minimum of 12 sessions, and some of them also received the booster sessions. The number of booster sessions was not registered.

### 2.3. Participants

Twenty-one families initially consented to participate in the PACT feasibility study. Of these, four families decided to stop during baseline assessments before the first PACT session, and just two received a few PACT sessions. Parents of the remaining 15 participating families were contacted and informed personally during child endpoint assessments or later by e-mail about participating in a qualitative interview concerning their experience with the PACT intervention. Parents were told that the purposes of the study were to apply the knowledge to future implementation of PACT and gain increased understanding of the effects of PACT. Two parents did not respond, and three declined the invitation. Hence, 10 families accepted the invitation to participate in the interview and signed an informed consent. Both parents were invited; however, it was imperative that the parent who primarily participated in the intervention was the same parent who participated in the interview. Parents did not receive any compensation for participating. In total, 13 parents participated in the 10 interviews: three couples, three fathers and four mothers (Table 1).

### 2.4. Data Collection

Data were collected through semi-structured interviews, as this is a valid method of obtaining information about parents’ experiences [22]. The flexibility and open-ended questions of semi-structured interviews produce rich and detailed data from the perspectives of the participants. The interview guide was informed pragmatically by psychological theories as well as reflections from Leadbitter et al. [20]. The first author (CEC) has formal training and practical experience with the PACT intervention and developed the interview guide, which was revised and edited by CA, TEG and MV. To understand parental attitudes towards their child being diagnosed with autism, a question regarding the parents’ reaction to the diagnosis was asked. Other questions originated from theories of the attachment system [12,23], the caregiving system [15] and the mentalising theory [16,24]. Several questions aimed to openly illuminate other relational aspects of the family and/or negative effects of PACT. Finally, questions regarding the feasibility of the intervention were asked in addition to the natural flow of the conversation, and prompts were used to elicit a more detailed description. See Table 2 for an overview of the themes and main interview questions. For a full translation of the interview guide, please contact the first author.

Through the process of collecting, analysing and reporting the data and results, the authors were aware of their positionality towards PACT and the possibility of positive bias. Three authors (CEC, PHT and MBL) were involved in both the DAN-PACT feasibility study and the currently ongoing DAN-PACT trial. Two authors (CA and TEG) were involved in data collection in the DAN-PACT trial. RJ was not previously involved in PACT research and could facilitate a critical view on any positive bias. As the first author had previously performed assessments during the feasibility study with some of the participants, the interviews were performed by one of the other authors who had no prior contact with the study participants. Parents knew that the interviewer was associated with the DAN-PACT research, but they had not previously been in contact. All interviews were performed in Danish by CEC (4), CA (4) or TEG (2) in June, July and August 2023. Pragmatically, it was decided to let the parents decide whether the interview should take place online, face to face in the clinic or in their homes, based on convenience. Six interviews were administered face to face, five in homes and one in the clinic. The remaining four interviews were administered online. All interviews were audio-recorded and transcribed verbatim by CA (4) and CEC (6), and the mean duration of the interviews was 67 min (range 45–115 min). As the interviews were conducted in Danish, all subsequent quotations are translated to English. The translations were not verbatim but true to what was said [25].

### 2.5. Analytic Strategy

**Figure 1:**
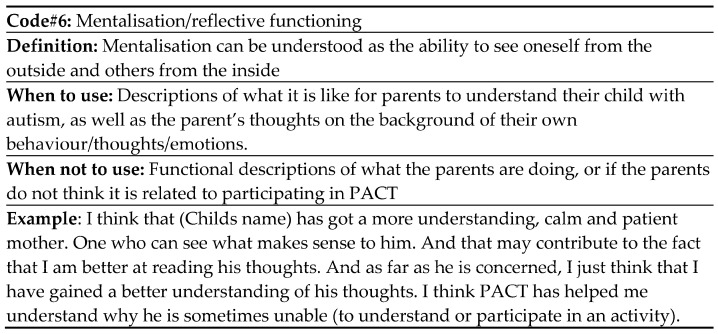
Codebook extract for the code relationship.

Analysis was inspired by an 8-step prescriptive approach to abductive thematic analysis by Thompson [26]. The transcribed interviews were colour-coded for related and similar phrases and meanings. All the codes were specific and concise. After two rounds of coding, a codebook was developed which labelled the different codes to increase the verifiability level (see e.g., Figure 1). The final step of coding using the codebook was performed by CEC, while three of the interviews were double-coded by CA. Coders (CEC and CA) then met with RJ to discuss potential themes. Codes were clustered into overarching themes, which could explain the phenomena. Following an abductive research approach, clustering and explanation of themes were guided—but not determined by—theoretical understanding [26,27]. Practically, this means that the theories of attachment, mentalisation and caregiving will be applied to the results section. Theorising was carried out during the fifth step. This step allows for cognitive engagement with data and theory in parallel to produce theoretical conclusions [26,28]. These theoretical conclusions will be further elaborated on in the discussion section. Step 6 in Thompson’s approach, *Comparison of Datasets*, was omitted in our analysis [26]. All authors took part in the final two steps which described the display of data and reporting of the results [26].

When analysing the study’s feasibility aspects, a more descriptive approach was used, as it did not make sense to make the same theoretical reflections regarding these aspects. This means that we did not use the fifth step of theorising concerning the feasibility theme in the analysis.

### 2.6. Ethical Considerations

The PACT feasibility study was registered at the Danish Data Protection Agency (Journal no.: 21/36108) and was reviewed by the local ethics committee which, according to Danish legislation, decided that they should not approve the feasibility study. Specifically, this qualitative interview study was not subject to the legislation concerning the committee law in Denmark due to its nature (not including human biological material). Statement: “*The following must not be reported: Health scientific questionnaire surveys and interview surveys which do not include human biological material* (Section 14, Section 2 of the Committee Act).”. No other ethical committee systems exist in the Danish region where the study could be ethically reviewed.

All data were kept in accordance with the regulation of the European Union regulation, i.e., the General Data Protection Regulation. Anonymity was ensured for the participants both in the transcripts and in the reporting of the results. In the transcripts and quotes, names have been changed or removed to protect anonymity. Participation was voluntary and participating parents were informed that their participation did not have any consequences for further treatment of their child. The interviewed participants all signed an informed consent form. The researchers were aware of how the study could potentially influence the family and took precautions to minimise any negative effects.

## 3. Results

**Figure 2:**
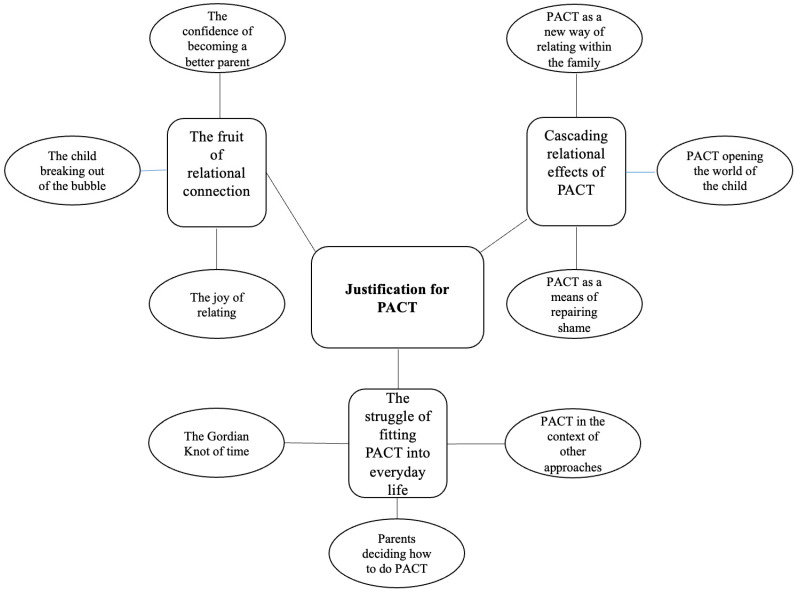
Themes and subthemes.

Three main themes connected by the main idea of “Justification for PACT” were derived from our data (Figure 2). The first theme concerned the feasibility aspects of PACT, while the remaining two themes concerned the relational aspects. “Justification for PACT” is understood as a tipping point of when beneficial relational effects outweigh the efforts it takes to participate in PACT.

1. The struggle of fitting PACT into every day life
2. The fruit of relational connection
3. Cascading relational effects of PACT

From each theme, three subthemes were derived.

### 3.1. The Struggle of Fitting PACT into Everyday Life

This theme concerns the feasibility of the PACT intervention and consists of three subthemes: The Gordian knot of time, Parents deciding how to do PACT and PACT in the context of other approaches.

#### 3.1.1. The Gordian Knot of Time

Time was addressed by all parents. Parents felt conflicted about whether the timing of the intervention was suitable as it came such a short time after the child’s psychiatric assessment. The parents experienced this time period as stressful and indicated that it held emotions such as grief, shock and acceptance, as well as structural changes to the family life.

*Busy around the time of the diagnosis. New institution, support pedagogue and case manager. So that’s pretty much the only thing I would say that it, PACT took a lot of time in a period when we had a lot of other things too.*

Fitting PACT into a time of structural change and emotional preoccupation was both challenging and helpful, as PACT provided professional support. One mother felt like the help provided by the PACT intervention compensated her for the time she invested in PACT:

*There is the time spent that can be a disadvantage. Because just that we had to take some time out and not work. But I still feel that we have gained a head start compared to how much time we could have otherwise spent figuring out how we should approach this diagnosis. When the whole thing is seen in that context, I think that it actually gave a really good extension of the fact that we had just been given that diagnosis, and then entered such a procedure. Although it seemed unmanageable when we were just about to start.*

For some parents, their emotional preoccupation inhibited their participation in PACT, as described by this mother about her partner:

*I think it has been difficult for him (the partner) to accept that he (the child) has autism. So, in that way, he (the partner) has kept it at a bit of a distance.*

All parents agreed that PACT was time-consuming. Scheduling time to participate in the sessions and doing the daily training at home was a struggle, as all the parents were working and daily life with a child with special needs already consumed time and energy. Some parents described how some PACT therapists would plan sessions at night or during holidays to compensate for the busyness of daily life. A few parents were compensated financially by the municipality for the time they had to take off from work because of their child, which was mentioned as a significant relief.

In addition to being time-consuming to participate in PACT, the parents also highlighted that it was stressful and that they felt pressure to practice and produce what they would consider a “good” video for the next therapy session. One father described how he experienced the mother’s stress before submitting a video:

*The only downside, I guess, was that right up until you had to submit a video, you couldn’t figure out which one to choose. You were just under a little pressure there sometimes. (…) It (the video) suddenly became a stress factor.*

Some parents described how changes in their child’s need for interaction suddenly demanded more of their time and attention.

*It was easier before [PACT]. We were not as tired as parents. After all, we used to have a boy who just needed some food, and then otherwise took care of himself. Now he expects some kind of interaction and play and all that.*

In one family, both parents had to be on sick leave with stress for a period of time because of the substantial development their son went through because of PACT.

*You are sitting across from two people who have practically collapsed with stress. And David, because he suddenly got language that he didn’t have before, he was suddenly able to put pressure on us in a different way than he ever could before.*

In spite of the strain on their health, these parents were grateful for the development their son went through, and for them, the positive consequences outweighed the negative effects.

#### 3.1.2. Parents Deciding How to Do PACT

A few parents had a high fidelity to the intervention and practised every day for 30 min. Others expressed a more eclectic approach and did whatever suited their family.

*It (the home practice) was realistic most of the time, but there were some days when we had to skip it. And then we just skipped it.*

*If I have to be 100% honest, and I have to be, then we could definitely have done more to practice more consciously. So, when I say that, of course, it can be used in many different contexts, but when I look back on the process, I wish we had had the energy to do it more—that is, practice it more between sessions.*

Some parents expressed dissatisfaction with the positive attribution of the PACT method and asked their therapist to point out every essential aspect in the videos, even the less positive.

*We had a pre-meeting with our PACT therapist about how to learn best. And I learn best by admitting my mistakes.*

*You can tell these psychologists yourself how you would like your feedback. Whether it should be direct or whether they should wrap it up a bit—so that you choose for yourself which level you want to talk about things at. And I kind of chose that she had to be open to me and direct, but still not too many, for example, not only negative things. I also wanted to be successful for myself.*

Some parents actively chose PACT online, whereas others mentioned it was a decision made by the therapist or the local clinic. However, all parents who received PACT online expressed contentment with the online sessions both due to saving time and the experience of the therapy being suitable online.

Some of the families decided to stop the therapy before all 18 sessions were completed. Different reasons were given for this, e.g., structural changes in the family, child needing somatic treatment, or a father experiencing the family did not need PACT therapy any longer since his son wanted to stop:

*One day, he (the child) asked: “Do I need to be filmed anymore, because I speak well now?” So he was not interested in being filmed anymore, and we told our therapist that, and she just said, “Fine enough, if David doesn’t want any more, that’s where we have to end it.” So, we did.*

All parents reported still using the principles of PACT in their daily interaction with their child, though some were more conscious about the interactions than others.

*Now you think it should be easy, but it hasn’t been for such a long time period we practiced (PACT) that we are now able to do the PACT strategies 100% perfectly. It is more difficult now to prioritise it. (…) It has become more of a part of everyday life, the thing about when he says a sound or something, we repeat it.*

Some parents also expressed worry about losing their applied PACT skills and suggested ongoing PACT booster sessions for them as individual families.

#### 3.1.3. PACT in the Context of Other Approaches

When talking to the parents about the goodness of fit of the PACT intervention with other approaches offered in community or educational settings, it was evident that the offers these families received for help were sparse. For example, none of the families had been offered other manualised interventions; however, some of the children were receiving interventions by speech therapists. All children were in age-appropriate institutions, e.g., day-cares, kindergartens or schools, daily. For most of the children, their institution was specialised in working with special needs broadly, or specifically with autism.

*No, we have not had any other treatment at the same time [as PACT]. After all, he has gone to kindergarten, and we have talked to them a bit about that (PACT), when they had troubles with him. But there has been nothing that conflicted [with PACT].*

Most parents described PACT as a helpful addition to other approaches offered in community or educational settings that focused more on the deficiencies of the child and not positive development.

*Well, because when you have a special child, you have many meetings where the only thing that matters is Nickolai’s deficiencies. We have talked a lot about all the things he couldn’t do in the first four and a half years of his life. And you had to get used to all that at the beginning [of PACT], and suddenly talk about all the good things. So, I think that was actually very nice.*

A few parents described exceptions where they were confronted with other approaches that did not align with the PACT strategies. One family experienced having to reject the behavioural-based method of a speech therapist from the child’s preschool who was not implementing PACT strategies:

*And if there’s one thing I can’t do, it’s doing… well, what we can’t do is dog training with a child like that. One, I think it’s too American. Two, it doesn’t suit who we are as people at all. And it was actually only when we said to her at one point, “Try to listen. You simply have to back off, and then let’s do what we have to do with David.”*

### 3.2. The Fruit of Relational Connection

This theme consists of three subthemes: the confidence of becoming a better parent, the joy of relating and “the child breaking out of the bubble”. Overall, the theme describes how parents experienced the PACT intervention creating a process of changes within the parents themselves and how the changes initiated an increased experience of enjoyment in interaction from both the parents and their children. Finally, how the positive experiences from the interaction with the parents motivated the children to communicate is described.

#### 3.2.1. The Confidence of Becoming a Better Parent

All parents, regardless of their initial caregiving strategy, expressed how PACT made them change their attitudes and behaviours towards their child and supported changes in themselves. Parents described an increase in both their sensitivity in caregiving [15] and parental mentalisation [17]. One father described an improved understanding of the child’s mental state and also reflected on the self both developmentally and as an agent of change.

*It is probably the development of oneself. I think that you get the most development yourself because you understand everything that is going on better and understand your own child better and know how to be more patient and succeed in things, instead of someone having to feel that they are failing at something.*

Most of the parents described the PACT intervention as a process of changing themselves. As the parents were implementing PACT strategies by observing their child, achieving mutual attention and being more sensitive and synchronous in their communication, they were simultaneously acquiring an increase in parental mentalisation and more sensitive caregiving [15,17]. One mother expressed how the increase in parental mentalisation makes her feel like a better parent:

*We have always loved Nickolai, and in that way, we have not become better parents. But we have gotten to know him better, and when we get to know him better, we become better parents. Because then we can better adapt our lives to him, or what to say, right? Yes, we would do that before too. We were just groping around a bit blindly there, so I think we have just got a bit of direction now.*

Some of the parents experienced that the change of self was difficult.

*It was also a way of working on myself, which was really hard. Because when you play with your child one-on-one, you also expect a certain kind of interaction.*

However, most parents were highly motivated to make an effort, especially this mother:

*There, I think I just felt like, okay, but if it helps a little, I’ll sacrifice anything in the world.*

Parents often described the process of change with an example from playing with their child, and an increased understanding and acceptance of the child’s autistic traits. An example is this father:

*When Emil doesn’t do exactly what you might like, you try to push him in the direction you think is normal. I kind of stopped doing that. So, that, so, yeah, that’s what I’ve stopped doing. Then there are still times when you think it’s annoying to play this. But basically, kind of accepting the games on his terms and kind of just being a part of it and not feeling like it has to be a certain way to be right.*

All parents had experiences that supported an improvement of the parents’ caregiving strategies and parental mentalising abilities through the PACT intervention [15,17]. This father was asked about the most important changes for his son:

*He may also now have a father who is better at understanding what he wants, and yes, sometimes it is difficult to understand what he wants, but then you try to follow along, or just do what you can.*

Even though the parents described PACT as having an immense impact, it was not described as solving all problems related to interactions between parents and their children with autism. Parents all come into parenthood with a story, attachment system and personality that provides them with different abilities to adapt to the behaviour and development of their child [11,15]. Some parents described feeling helpless in their caregiving strategies. This could be the case when the children had meltdowns, or like this father, in the context of repetitive behaviour, described. In these situations, the parents felt inadequate as parents.

*My problem is that I really want to be able to tolerate it (repetitive sounds), but I just can’t. It actually makes me feel really powerless because I have a hard time dealing with that, and it’s something I know I just have to deal with and sometimes we also have a talk about did I cross the line too much there, or was it okay for me to do that? I have no feeling for it at all. I just have no feeling for that part.*

#### 3.2.2. The Joy of Relating

The concept of mutual enjoyment is known from attachment research and is characteristic of parent–-child dyads with sensitive caregiving and securely attached children [12,15,29]. Mutual enjoyment occurs when the relationship becomes genuine, and both parent and child experience the joy of being together without agendas of learning or other functional concepts. Mutual enjoyment deepens the attachment relationship, and when the parent–child relationship lacks mutual enjoyment, it fosters negative affect [29]. Some parents described how acceptance of their child’s characteristics was important to achieving mutual enjoyment. This mother explained how her improved parental mentalising capacity toward her child helped her enjoy the interaction with her son [17].

*Yes—understand him better. Can lean back a little more. But I think you set aside your barriers and expectations and then you just enjoy the moment you’re in. I think it’s more that, as far as I’m concerned (…) But the most important thing for me was being able to communicate with my son.*

Some parents described that when mutual enjoyment was achieved in the interaction with their child, both parent and child were naturally encouraged to stay longer in the interaction and resume interaction at another opportunity. This father beautifully described a play situation with mutual enjoyment where time ended up not being important:

*But for fun, I build a lot of Legos. I like that it gets into a rhythm with it. Where we can sit and play with it. He can sit and play a bit with his, and then I can help him a bit, and then I take a bit of what he’s building. You can imagine such a situation, where it does not make such great demands on us, where we kind of just do what we want. (...) like in everyday life at home, it’s really, really nice. Cozy.*

Some parents described how changes in the attachment system and parental mentalisation were due to a bi-directional improvement [17,30]. The parents improved their skills of understanding their child’s thoughts, feelings and intentions, but their children also became easier to understand because of improvements in their communication skills and motivation. Some parents experienced that the increased sensitivity in parental caregiving initiated a closer emotional bond and awakening of mentalising capacity in the child, who started to recognise the parents as individual beings [23,31]. This father described how he used to feel more like an object in the eyes of the child and now has become a subject:

*We just weren’t there before. Mother was a pair of breasts. And I was a claw [that picked things up for the child or gave him what he wanted]. So, more or less. That’s what we were used to, after all. And now he wants to play, and he wants to tumble, and he wants to kiss. You saw even when we said goodbye now. Then he wanted all that close contact and all that. He didn’t mind close contact before. But it wasn’t because it was us. (...) No, it wasn’t because we were his parents, and he had some kind of relationship with us. It’s something new. That he has built a closer relationship with us and his sister. Because he has begun to see us as individuals, and not just something that was there.*

Parents reported whether they perceived any change in their children’s attachment behaviour in the contexts of comfort-seeking and reuniting with their child after being apart [23,29,30]. A few parents did not experience any change, as their children always had sought comfort and reacted positively when reunited. Some parents were not aware of if comfort-seeking had changed from before PACT, while others noticed a substantial improvement. Some parents, like this mother, described a change in comfort-seeking behaviour after implementing the PACT intervention:

*So you couldn’t (comfort him). So he could fall and hit himself, and then there was nothing. Where... If we tried to comfort him, we couldn’t comfort him because he pulled away. He actually almost got mad when we tried to comfort him. And we can do that today. We may be allowed to come to him there. (…)Yes, I think we can comfort him now. It has become better.*

Some parents described a change in the child’s reaction to them when they were reunited. Like this father who described a change from no reaction at all when they were reunited before the PACT intervention to this:

*And then he comes over, and then he has to have the world’s biggest hug. And then it’s the deepest “HIIIIIIIII!”. It’s all just riding on him. Yeah, he’s pretty crazy.*

Parents also described attachment behaviour from the children which demonstrated increased proximity-seeking behaviour and emerging stronger emotional bonds:

*I think in general, what you want is that closeness with your children. Just being close somehow. At least he comes, that is, when he needs it, he comes and looks for it himself.*

While it was not evident that attachment behaviour had improved in all the interviews, all parents found the PACT strategies helpful in relating with their children.

*This has meant that I have started to be able to play with him. Where it has, well where we have just existed in the same room before. And it has been nice. It has been really nice to have this relationship with him. (...) It has resulted in a calm everyday life. Not because it was troubled before, but before this (PACT) we didn’t have the same interaction as we have now, so it’s a more social everyday life we have.*

Attachment relationships can be different between the child and each parent [32,33]. Some of the parents described this difference and experienced how being the primary parent in PACT improved their attachment bond with their child. One father described a change in how the child also sought him for comfort:

*Well, there has also been a tendency towards mother. But it has also become more, it has also become more equal. (…) Yes, generally how he seeks me. I mean, in the past, it has been like that, actually, he hit himself, for example, and he got upset, and then I picked him up, and then maybe mother came walking into the living room. So, he kind of turned away from me. It is not like that anymore.*

#### 3.2.3. “The Child Breaking out of the Bubble”

Several parents experienced and explained the child’s social–communicative improvements as breaking out of a bubble:

*Before, he could easily sit down in his room and play with something, like that and sit in his bubble with something. He rarely does that now. In fact, now he wants to involve the rest of us. Both us and little brother too.*

A few parents also described the development of early mentalising ability in their children and how it built the relationship with them [31]. This mother perceived that the child had awareness of himself coming out of a bubble, and this was creating the connection:

*Now he knows we are here because we do something for him, but also because we love him and because he loves us too, we assume, right? But I think in that way, it is building our connection that he also understands that he is out of that bubble.*

Parents described the child’s development as due to experiences of communication making sense and being enjoyable. The parents’ improved sensitivity and parental mentalisation motivated their child to communicate.

*Because as we have improved, Anton has found out how much it makes sense to communicate. And because he has found out that it makes sense to communicate, he has started. And then he has practiced. He has gotten better and better. And now he talks to us and gets to say what he wants and such. He is approaching us very much now. All the time actually.*

Some parents experienced their nonverbal children acquiring language:

*After all, he only started by saying, “help me”. It was his first after he lost his language and then it came back. “Help me” was his first little phrase. And then it just developed and he’s a stubborn boy.*

Some of the children with delayed language experienced an improvement in language skills:

*In other words, the communication skills are amazing. He is understandable in a completely different way. Also, just that I could be told that he only wants Lightning McQueen shirts. So ‘nice to know’.*

The following statement demonstrates how verbal language, on top of making it easier for the parents to practically understand the needs of the child, also had a function in strengthening the emotional bond between parent and child:

*In the first video, he is standing up by the windowsill and looking down at me, and there she (the therapist) says, if he could say anything there, he would say “I love you.” She was absolutely sure of that. Because he stood like that and looked for quite a few seconds, but stood like that and looked at where I was. And the last video I sent her, it was because we had talked about it, the first video, and it makes an impression on a mother when he couldn’t say “I love you.” And the last video I sent her, he directly says “I love you”.*

According to the parents, some of the nonverbal children were still nonverbal after PACT, but all of those children had acquired other communicative skills such as gestures and eye contact.

*It hasn’t changed that a lot, apart from the fact that, after we started PACT, there has been a lot more things like taking my hand and then pulling me to the kitchen if he wants something to drink. Or you know, he tries to take me to what he wants instead. So, there is more body language than there is verbal language anyway.*

Several parents emphasised that improvement in eye contact was caused by the PACT intervention. They described eye contact as important for communication regardless of whether the child was verbal or not. This mother related eye contact to a way of getting to know her child, which places eye contact as a potential facilitator of improving parental mentalisation [17].

*I actually think in relation to the eye contact from when we started PACT until today—it is miles away. And I’m quite sure that has something to do with PACT. So, we have a lot of eye contact with Laust, where he seeks it out a lot and we have a lot of contact. It has been a way for us to get to know Laust as he is.*

### 3.3. Cascading Relational Effects of PACT

The third theme consists of three subthemes: PACT as a new way of relating within the family, PACT opening the world of the child and PACT as a means of repairing relational shame. Overall, this theme concerns how PACT had cascading effects on improving relational aspects beyond the parent–child interaction. Changes were seen in other relationships within the family and between the child and other people outside the family; for example, how the PACT intervention and the relationship between parents and the PACT therapist had the power to repair feelings of shame in the parent, thus enabling the parents to engage in the relationship with their children.

#### 3.3.1. PACT as a New Way of Relating within the Family

Parents reported relational changes within the family that were beyond the relationship between the parent and child with autism. Seven children had a sibling, and all parents described some change in the relationship between the child with autism and the sibling. Changes were primarily positive with increased contact and interaction; however, one father described the new sibling relationship as creating some problems:

*It has changed from him being a very little brother that she didn’t really have to deal with (…) To now where he is a more equal sibling. That is to say, now there has started to be a conflict between them. It wasn’t there before. So, in that way it has definitely changed from him being an indifferent little brother to [him] being an equal sibling to some extent. They play together a lot. They have a really good time together.*

Some parents also described how the siblings benefitted from the parents using PACT strategies with them.

*You can say that what we have done with David, that is, his little sister has also benefitted immensely from it. Because we have used some of the same things on her as well, quite intuitively. Completely intuitively. Someone we know is a speech therapist and some of what she says is that she (the sister) has language that is approx. a year and a half ahead of where she should be. And that came from that (PACT), I have no doubt at all.*

Several parents described how grandparents and other relatives were important to the well-being of the family. Some parents described how relatives could serve as witnesses who affirm parents in the positive developmental change in the child and how this enhanced their motivation for the PACT intervention. All parents talked about the relationship between grandparents and their grandchildren. Some grandparents were not involved in PACT. A few had applied PACT strategies in communication with their grandchild with success, and a few parents, like this mother, wished the grandparents would engage in PACT strategies.

*I have had a conversation with my mother and told her that it could be nice if they got involved. So, my answer is really that no, there aren’t really others who have thrown themselves so much into it. But it’s obvious, it’s simply a matter of doing it. And it’s really simple things, but they’re just things you don’t think about.*

Some parents even taught PACT strategies to the grandparents, like this father who taught his parents:

*So, it’s one of those things about articulating what it is we’re actually doing. And what is it all about. Because they ask out of curiosity. Okay, what are you guys doing? We are doing this and that and it is really about us getting better at reading Anton, and mirroring him, and helping him to put into words what is going on. Okay, that’s understandable. Then it’s about trying to do the same.*

#### 3.3.2. PACT Opening the World of the Child

Some parents reported how their children applied their communicative skills to other relationships outside of the immediate family. This happened within the child’s institution and other contexts. Parents described how their child became able to relate and play with other children. Parents described this as caused by increased communicative skills; however, the change could also be partly due to an improved sense of security. Securely attached children will experience their primary caregiver as a secure base from which they can curiously explore the surrounding world and form new relationships without anxiety [23,29].

*Now they play just as much with each other, those children across the street. And it is also across age groups. (...) And it is because he looks up and sees that there are others. He wouldn’t have dreamed of that before.*

Some parents experienced that they could do activities like going to the grocery store or even amusement parks with their children. This was something that they could not do before PACT. This father described how his son expanded his possibilities in relation to strangers:

*But what I really wanted to say is that he can come out, and then sort of express some of those things. There was a lady one day who said hello to him, sitting in the car and then he says: “I love that lady” because she gave him the attention, he thought he needed at the moment.*

The generalisation of social–communicative skills of the children may have been reinforced by some parents sharing PACT strategies with the professionals in the child’s preschool, as this father describes:

*The dialogue we have had through PACT, and the things we have observed there, we have shared with the educators. So, they have been able to use some of the same things in their work with Anton. So, it has been really rewarding. It worked so well in terms of strengthening his social skills that all of a sudden, there was another child in relation to things like turn-taking and some of the other things that you also have to learn at one point or another. So, there has been a great deal of focus on social development and the institution that has worked with the things that we have worked with at home.*

#### 3.3.3. PACT as a Means of Repairing Relational Shame

Matters of shame in different forms occurred repeatedly in the interviews. Shame can be linked to feelings of being worthless, diminished and exposed [34]. Some parents reported feelings of shame at the fact that they have a child who is “not normal” or has “special needs”, implying that the diagnosis is caused by faults in themselves.

*Yes, it was tough, because I think that when he did not develop as I expected, it was because I compared him to a neurotypical (child). Up in my head, I still hadn’t adjusted, because I didn’t know what autism really was. (...) He goes to a special school. That way you still feel that defeat.*

When parents referred to their children as being “different”, it was often in the context of their children becoming more “normal” after going through PACT therapy, like this father who experienced shame and elicited desires to hide or escape [34]. The improvement through PACT had helped repair the shame.

*I had for a time before PACT almost developed a minor social phobia, because he behaved so strangely out on the playground, and now we just kind of just got to a place where it’s not a problem anymore.*

In this following example of social shaming, the feelings of shame in the father seemed to be repaired by the relationship he achieved with his nonverbal son when he used a PACT strategy:

*Although people can sometimes look at you strangely when you’re out in the grocery store and Mikkel says “uhuhuh”, and I also say “uhuhuh”, but then he laughs and smiles. (4)*

Some parents also expressed shame for not being a good enough parent [15,35]. This father felt useless, but through his participation in PACT, he gained confidence in his parenting skills.

*Well, that means everything. This means that I can do things and not just stand on the side feeling helpless and very useless. Because before, well, he was upset, so I could stand and wait for his mother to come, because I couldn’t be used. (...) I just think that it has been very important (PACT), and it has been crucial for our family to be able to be involved in this, I think. And I actually also think that it has helped our family relations in relation to the fact that others may have thought that we have done things wrong—that is, in the upbringing of David and things like that.*

Some parents described how their PACT therapist had an important role in guiding them through a chaotic time in their lives. For some parents, the therapist guided them through the transition and repairing shame by encouraging more sensitive caregiving [15,23], acceptance and understanding of the child’s diagnosis as well as symptomatology and understanding of the child and their own role as parents as they practised and improved their parental mentalising ability [17,31].

*I also think that PACT has helped us get through it well. We have been given the opportunity to put into words some issues and some of his behaviour trying to understand why he does what he does and trying to connect with him and so on. I think it has been really nice to have someone to go to when you have questions. I also looked forward to telling her (the PACT therapist) that he had been given a place in special education—and I celebrated things like that with her. That way, when you have so many sessions, a lot of other things happen. She (the therapist) has been a good anchor.*

**Figure 3:**
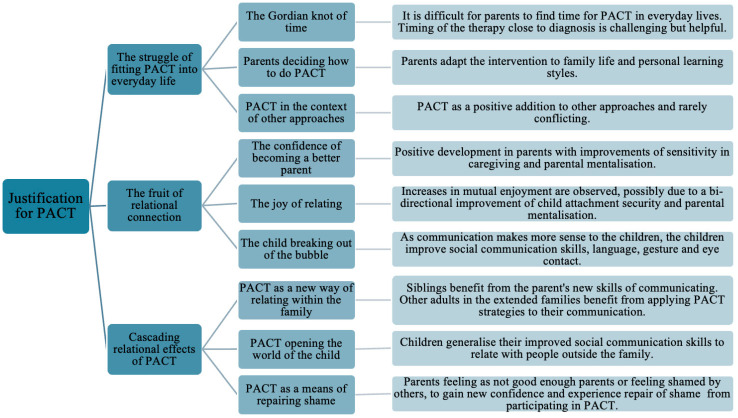
Summary of main findings.

Results are summarised below in a synoptic table (Figure 3) showing the themes, sub-themes and summary of main findings.

## 4. Discussion

This qualitative study uses a thematic analysis inspired by an abductive approach and contributes insights into the feasibility of the PACT intervention and mechanisms of relational changes informed by theories of the attachment system, the caregiving system and mentalisation.

### 4.1. Feasibility of the PACT Intervention

The feasibility of the PACT intervention in everyday life was considered challenging by parents. The challenges were related to participation in the PACT intervention close to the child receiving the diagnosis, having enough time to participate in the sessions, doing the home training, adaption of the family to developmental changes of the child, participation of the other parent and goodness of fit with other approaches offered to the child with autism.

Offering the PACT intervention close to the time of the child being diagnosed could seem inappropriate as some of the parents were either emotionally preoccupied and struggling to accept their child’s diagnosis of autism or occupied with structural changes of the child going to a new institution because of the autism diagnosis. However, the advantages of having guidance in this challenging period of the families’ lives could outweigh the disadvantages. Parents clearly benefitted from the validation and guidance they received from their PACT therapists. This empowerment of parents may be relevant for understanding the longer-term benefits previously evidenced in PACT [7,20].

The emphasis on time being a challenge was also recognised in the qualitative study from the UK [20]. However, this seemed to be more urgent in this Danish study perhaps due to all participants being employed, which is not atypical for a Danish family with younger children.

Some parents were aware that the intensity of their home training affected the pace of their child’s development. In families where children acquired the greatest developmental changes, a pattern of parents with high fidelity to the requirements of daily training at home was recognised. However, it was evident that all parents had trained as much as their individual lives and personalities permitted, and all parents experienced some sort of developmental and relational improvements in their children. High fidelity to the parent-mediated interventions for children with autism was in a systematic review recognised as associated with significant changes in the children’s social–communicative functioning [36]. There is a potential conflict between manualisation and individualisation of the therapy. This is seen when parents want to change the attitude of the therapists to be more direct about negative parental behaviour, and hence compromising the positive attribution of the behaviour from the PACT manual. However, the manual also emphasises the importance of adjusting the therapy to the learning style of parents. So, when the parents directly ask to have more nuanced feedback from the therapists, it would be right to do so.

Parents adapted the framework of PACT to their individual needs, values and wishes. Specifically, in regard to the primary and secondary parents participating in the intervention, the authors anticipated conflicts arising; however, hardly any conflict was experienced. As the families adopted the intervention, the other parents participated as was convenient for the specific family. Hypothetically, participation of both parents would expose the child to a more intense relational change; however, there was no clear indication of change being dependent on both parents being active.

This Danish study showed some structural differences compared to the UK study by Leadbitter et al. [20]. In Denmark, videos of parents interacting with their children were recorded at home, and the children did not accompany the parents to the therapy. This circumvented some of the troubles described by the UK parents of the children disturbing the therapy.

The goodness of fit of the PACT intervention with other approaches on offer in community or educational settings was mostly unproblematic and even beneficial. The positive attribution of PACT is assumed to enhance parental self-efficacy and strengthen them through the external focus on the child’s deficiencies. When parents experience the benefits of the PACT strategies, their PACT therapist should encourage them to share this with professional caretakers, teachers and relatives close to the child to create opportunities for positive cascading effects of PACT.

### 4.2. Mechanisms of Relational Change

**Figure 4:**
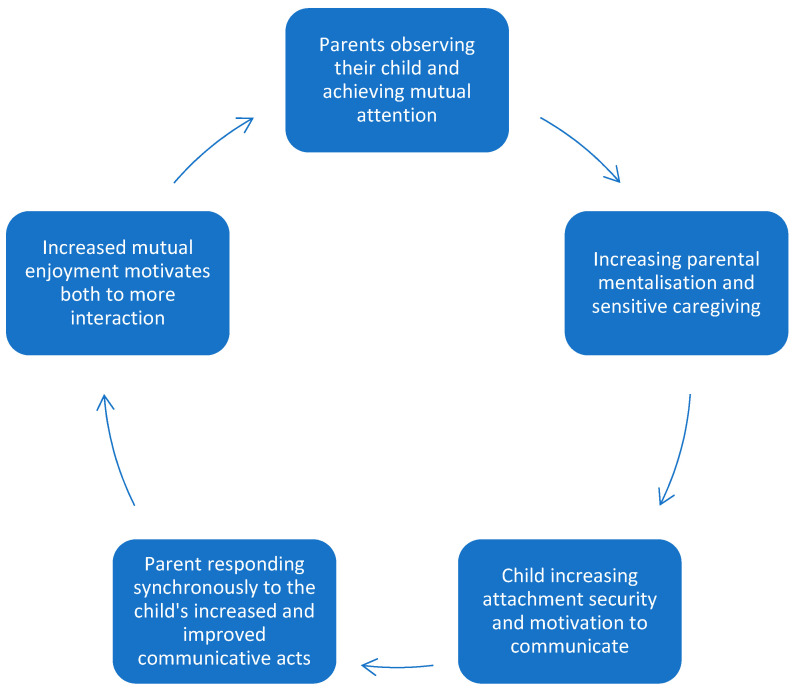
The positive cycle of relational change through the PACT intervention.

The finding that all parents experienced relational changes strengthens and confirms the results from both previous quantitative and qualitative studies in the PACT intervention [3,7,20]. This study contributes with an abductive approach of reflecting theoretically on the mechanisms of relational change by applying concepts of attachment, caregiving and mentalisation. A pattern of change initiated by the PACT intervention was recognised through the narratives (Figure 4). Specifically, during therapy, parents were taught to wait and observe their children and follow their cues in order to achieve mutual attention. By doing this, parents could understand their children better, leading to increased mentalisation and a more sensitive caregiving strategy. The PACT intervention could initiate at least two beneficial developments in the children: increased attachment security and development of communicative skills. Attachment security has been previously associated with parental sensitivity, and this association seems to be even stronger when the child has autism [37,38]. In this study, parents experienced an increase in their sensitivity, which was also recognised in previous studies of PACT [3,5]. There was also indication of some of the children demonstrating behaviour of increasingly secure attachment behaviour regarding comfort-seeking, reaction to the parent in reunion, curiosity in exploring surrounding contexts and creation of stronger emotional bonds to the parents. When the child interacted with their parents, who had adopted a new communicative style from PACT, the parents experienced the child as increasingly motivated to communicate, presumably as it made sense to the child (i.e., the child experiences a parent who responds in synchrony with them and a parent who understands them). Specifically, the child would begin communicating more and hence improve communicative skills such as language, eye contact and gestures. Consequently, they would be increasingly understood by their parents. Both of these beneficial developments would further strengthen the parent and child interaction, with an increase in child communicative initiation. The parents would increase their abilities in meeting their child with synchronous interaction and generalise their PACT strategies to other everyday activities than the training. This positive pattern of relating would lead to increased mutual enjoyment in the interaction which would motivate the parent–child dyad to spend more time together, giving parents better opportunities to observe and get to know their child and thus closing a positive cycle of relational change, as shown in Figure 4.

Parents also described a cycle pointing out of the parent–child relational system. Specifically, when the child acquired increased attachment security, they would consider the parent a secure base [23,29], while with improved communicative skills, the child would increasingly interact with people other than the parent. This would contribute to further communicative improvement and mutual enjoyment in relationships other than those with their parents.

Another aspect not indicated in Figure 4 is the possible development of mentalising in the child’s ability. The child was perceived to be conscious about the change in increased connection with others. This was also observed in how the child perceived their parents from objects to subjects. These processes both describe mentalising ability development [16] and seem to be a significant accomplishment to obtain through an intervention. The development of mentalising ability in children with autism is aligned with the theory of the double empathy problem, describing how people with autism have abilities of empathy with each other, in the same way as people without autism have empathy with each other [39]. The double empathy problem is two-sided and occurs when a person with autism and a person without autism should be empathetic to each other. The theory of the double empathy problem also claims that often people with autism have to learn to be more empathetic to adjust to a social world on the premises of people without autism [39]. It could be that PACT is an intervention working precisely around this problem, trying to increase the parent’s understanding of their children with autism, and possibly facilitating the development of mentalising ability in the children. In this study, it is not evident whether the children are actually developing some mentalising ability or the parents perceive the development of mentalisation in their child through their interpretation of the child’s interaction. Either way, the changes in the possible development of early mentalising ability made a positive difference in the parents’ interpretation of the interaction.

As the theories of attachment, caregiving and mentalisation contribute to the understanding of the relational mechanisms of change through the PACT intervention, this study also contributes to the theories themselves by describing empirically how the theories unfold in daily living. Recognising that the PACT intervention aims at improving parental mentalisation, and empirically confirming how this affects the social and communicative development of the child, also highlights the significance of parental mentalisation. The focus on the relational improvements in this study also emphasizes a normative and transdiagnostic effect measure, in contrast to a focus on reducing autism spectrum symptomatology.

The therapeutic alliance and concept of shame were not addressed directly in our interviews. However, these matters emerged continuously, and it was evident that they had an impact on the relational aspects of our study. A previous study shows evidence of parents having children with autism experiencing more shame than other parents [40]. The shame was based on the perception of the parents themselves failing as parents and shame regarding the child’s atypical behaviour in social contexts [40]. Both of these feelings of shame were reflected in our results. As shame comes with feelings of wanting to hide or escape, it is likely that some of the parents were not ready to participate in PACT and avoided interaction with their children as they experienced intense shame [34]. Though it was not named “shame” by Leadbitter et al. (2020), experiences of feeling judged and criticised in social situations were described. As recognised in the results of this study, the therapist validating the parents and the prominence of the parents’ own development and capabilities as parents would repair the feeling of shame. The repair of shame in the therapeutic alliance is described as a process of change in the parents themselves, achieving more sensitive caregiving, feeling empowered and feelings of becoming a better parent. Themes of the impact on the parents themselves were recognised from previous qualitative research on parental perceptions of autism interventions [41,42,43,44]. This study adds to the body of evidence by demonstrating how these parental changes may repair shame through the therapeutic alliance.

### 4.3. Limitations

This qualitative study was concerned with the feasibility aspects of the PACT intervention and relational changes. It was not possible to address all issues mentioned by the parents, e.g., subjects of technical difficulties were omitted, as they were of a more local concern. In the Leadbitter et al. (2020) study, it was described that the UK PACT therapists were educated speech and language therapists, while in Denmark the educational backgrounds of the PACT therapists were diverse. This issue was not addressed in our study. All the Danish PACT therapists had clinical experience with children with autism and their educational backgrounds were nurses, psychologists, psychiatrists or pedagogues.

Four families from each clinical setting were offered participation in PACT. This involved families that had anticipated resources and capabilities to complete the intervention and required assessments. Thus, recruitment to the feasibility study was not random. Initially, the authors decided not to interview parents who only had a few PACT sessions, thinking that they would not be able to contribute with new information about the effects of PACT. Upon later reflection, it could have been illuminating to understand their perspectives. Additionally, the participating parents primarily being educated parents with higher incomes is recognised. All but two children lived with both parents, which may be representative of younger children in Denmark. However, none of the children in single-parent households had regular contact with the other parent, which would have imposed further problems of implementation of the PACT intervention. Parents unable to speak sufficient English or Danish in order to participate in the intervention were excluded from the feasibility study. This made the sample culturally homogenous. Only parents of boys participated in the qualitative study. Only three girls participated in the feasibility study, and unfortunately, these families left the study during baseline assessments or did not wish to participate in the qualitative interviews.

This qualitative study did not address the question of data saturation, and all thirteen parents from our feasibility study who consented to participate were interviewed. The authors’ impression was that new themes did not arise within the last two interviews, and it is unlikely that additional interviews would have contributed significantly.

The parents may have been positively biased in the reporting of their experiences with the PACT intervention, as some of them wanted other families in the same situation to be given the same opportunity of receiving PACT. This was seen as several parents expressed a wish for the PACT intervention to be offered to all parents having children with autism. By using a researcher whom the parents had not previously met during the feasibility study interviewing the parents, minimisation of positive bias was attempted.

### 4.4. Clinical Implications

The findings of this study lead to several clinical implications for PACT therapists and clinics to consider.

Concerning the feasibility aspect, it became evident how essential the individualisation of the therapy is, as the parents have the freedom to adapt the framework of PACT to their individual lives in collaboration with their PACT therapist. The authors suggest that the therapist maintain the developmental steps and principles from the PACT manual, while being ready to individualise whenever possible the learning style, frequency, administration online or in-person, time of the day of sessions, and even number of sessions to the specific family’s needs.

As it is evident that home training is essential to mediate relational change and social–communicative improvement in the child, PACT therapists should encourage parents to follow the training at home on a daily basis. The therapists should not impose feelings of shame or guilt on parents when they are not succeeding. Rather, the therapists could support the parents in exploring how the training could fit into their schedule without too high a price on daily family life.

It is recommended that PACT therapists talk to parents, when both do not participate in the PACT intervention, about the role of the other partner (or close caregivers). When the other parent is not involved, the therapist could collaborate with the primary parent to investigate possible ways of involving the other parent.

Some of the parents experienced benefits from applying the PACT strategies to siblings not diagnosed with autism. This raises the question of whether PACT could be used as a transdiagnostic intervention for children with communicative disabilities other than autism.

To ensure the continued effects of the PACT intervention, clinics offering PACT may consider ongoing, e.g., yearly follow-up booster sessions as new challenges will occur as the child ages. This could be offered on an individualised basis as long as it makes sense to the family and the PACT therapist.

The PACT therapist could recommend that participating parents share with extended family and educators PACT strategies that worked for their child and encourage them to use the same strategies. This has the potential to enhance the positive cascading effects of the intervention.

Through the interviews, it was evident that the PACT intervention promotes sensitive caregiving in parents, and that both parents and children benefitted from this. Often, parents of children with autism are recommended to support their children through structure, predictability and visual support, all examples of more functional caregiving. This raises the question of whether autism symptomatology and professional guidance both prompt parents to acquire a more functional and not-as-sensitive caregiving strategy. It is more difficult to be flexible around a child who requires structure. When guiding parents to enhance structure, it may be essential to not forget sensitivity. Parents could benefit from clinicians increasing awareness of the strength of sensitivity in caregiving, guiding parents to observe their child to understand, respect and follow both their verbal and non-verbal cues and not just recommend visually reinforced structure and predictability.

Clinics offering the PACT intervention should be aware of how developmental change in the child affects both the family dynamics and the possibility of negative effects. The PACT therapists should be sensitive to any need for additional support for the family to prevent reactions of stress. Acknowledging the importance of and caring for parental well-being is essential to promoting positive outcomes for both children with autism and their parents.

### 4.5. Future Research

This qualitative study indicated that changes may be recognised in the children’s attachment system. Future research should investigate if PACT has the ability to change the attachment system in children with autism, as this was seen with another parent-mediated intervention [14].

The study did not address the questions of the impact of parental attachment representation. As parents’ own attachment is related to their ability to mentalise with their child, it is likely that the parental attachment representations would affect parents’ application of the PACT intervention [17]. Securely attached parents are more likely to adopt a sensitive caregiving strategy and demonstrate more flexibility and confidence in others [15,29]. Hence, these parents would more effortlessly benefit from the therapeutic alliance. Future research could investigate the significance of parental attachment representations in relation to parent-mediated interventions for children with autism.

Additionally, the following areas of future research in the PACT intervention could be of interest:

- Examine the impact of parents’ fidelity to implementation on outcomes of the intervention.
- Examine the impact of the profession of the PACT therapist on outcomes of the intervention.
- Implementation of the PACT intervention for children with divorced parents and culturally diverse families.
- The significance of both parents being active in the intervention.
- The influence of the cascading effects when the extended family or the child’s educators learn and implement the PACT strategies in their interaction with the child.
- The effect of PACT as a transdiagnostic intervention applied to children not diagnosed with autism but having social–communicative disabilities or other developmental disorders.

Furthermore, future qualitative research could, by using other qualitative methodologies, lead to new discoveries about the effect and mediators of parent-mediated interventions such as PACT. This could be carried out by using methods of observation of the child at home and in educational settings.

## Figures and Tables

**Table 1:** Participant Characteristics.

Family Characteristics		*N*
Child living with	Both parents	8
	Mother	1
	Father	1
Language spoken in family	Danish	9
	Mixed	1
Household income	<40,337 EUR	0
	40,337–67,230 EUR	1
	67,230–94,121 EUR	3
	94,121–121,013 EUR	2
	121,013–161,351 EUR	2
	>161,351 EUR	2
Parent Characteristics		
Age at time of interview		33.2 years average (range 28.3–40.2 years)
Born in	Denmark	9
	Other	1
Education	Secondary school or less	0
	Short education	3
	Middle education (e.g., bachelor’s)	5
	Long education (e.g., master’s)	5
Employment	Employed *	13
	Unemployed	0
Psychiatric disorder	Yes	0
	No	13
Parent’s status	Mother	7
	Father	6
Child Characteristics		
Age at time for baseline PACT feasibility		Mean 3.89 years (2.65–6.31 years)
Sex	Male	10
	Female	0
Born in	Denmark	10
	Other	0
Genetic Disorder	Yes	2
	No	8

* Some parents had to take time off from work to take care of their child with autism, which was paid for by the municipality.

**Table 2:** Main Questions of Interview.

Theme	Question
Participation in Paediatric Autism Communication Therapy (PACT)	Try to talk about how you got involved in the PACT project.
2. Reaction to the diagnosis	How did you react when your child received the diagnosis?
3. The experience of PACT	Could you tell me what it was like for you to be involved in delivering the PACT intervention to your child?
4. Caregiving strategies	What is most important to you in terms of how you take care of X? (Any change?)
5. The Family	Can you tell me how participating in PACT has affected the whole family?
6. Mentalisation	What is it like for you to understand what X thinks and feels? (Any change?)
7. Relationship in the dyad	Can you tell me if participation in the PACT intervention has meant anything for your relationship with X?
8. Attachment behaviour	How does your child react to seeing you when you have been apart? (Any change?)
9. PACT now	What do you think PACT means for your daily life now?

## Data Availability

The data presented in this study are available upon request in Danish from the corresponding author due to ethical reasons.

## References

[1] Lord C., Traolach S.B., Tony C., James C., Guillaume D., Thomas F., Emily J.H.J., Rebecca M.J., Andrew P., Matthew W.S. (2020). Autism Spectrum Disorder. Nat. Rev. Dis. Primers.

[2] Fombonne E., MacFarlane H., Salem A.C. (2021). Epidemiological Surveys of Asd: Advances and Remaining Challenges. J. Autism Dev. Disord..

[3] Green J., Tony C., Helen M., Catherine A., Vicky S., Pat H., Ann L.C., Kathy L., Kristelle H., Sarah B. (2010). Parent-Mediated Communication-Focused Treatment in Children with Autism (Pact): A Randomised Controlled Trial. Lancet.

[4] Conrad C.E., Marie L.R., Jeanett F.R., Birgitte H.P., Christoffer B.K., Simon T., Cathriona C., Marlene B.L., Mina N.H. (2021). Parent-Mediated Interventions for Children and Adolescents with Autism Spectrum Disorders: A Systematic Review and Meta-Analysis. Front. Psychiatry.

[5] Aldred C., Green J., Adams C. (2004). A New Social Communication Intervention for Children with Autism: Pilot Randomised Controlled Treatment Study Suggesting Effectiveness. J. Child Psychol. Psychiatry.

[6] Sandbank M., Kristen B.-B., Shannon C., Margaret C., Kacie D., Jacob I.F., Jenna C., Susanne A.A., Sweeya R., Prachy M. (2020). Project Aim: Autism Intervention Meta-Analysis for Studies of Young Children. Psychol. Bull..

[7] Pickles A., Ann L.C., Kathy L., Erica S., Rachel C.-F., Hannah T., Isobel G., Jessica L., George V., Sarah B. (2016). Parent-Mediated Social Communication Therapy for Young Children with Autism (Pact): Long-Term Follow-up of a Randomised Controlled Trial. Lancet.

[8] Carruthers S., Andrew P., Tony C., Helen M., Ann L.C., Vicky S., Patricia H., Rachel C., Erica S., Hannah T. (2024). Mediation of 6-Year Mid-Childhood Follow-up Outcomes after Pre-School Social Communication (Pact) Therapy for Autistic Children: Randomised Controlled Trial. J. Child Psychol. Psychiatry.

[9] Green J., Kathy L., Ceri E., Lauren T., Heather L.M., Sophie C., Kirsty J., Carol T., Matea B., Sophie L. (2022). Combined Social Communication Therapy at Home and in Education for Young Autistic Children in England (Pact-G): A Parallel, Single-Blind, Randomised Controlled Trial. Lancet Psychiatry.

[10] Grzadzinski R., Carr T., Colombi C., McGuire K., Dufek S., Pickles A., Lord C. (2016). Measuring Changes in Social Communication Behaviors: Preliminary Development of the Brief Observation of Social Communication Change (Boscc). J. Autism Dev. Disord..

[11] George C., Aikins J.W. (2023). Developing a Secure Base in Family Intervention: Using the Adult Attachment Projective System to Assess Attachment in Family Relationships. Front. Psychol..

[12] Bowlby J. (1969). Attachment and Loss.

[13] Fardoulys C., Coyne J. (2016). Circle of Security Intervention for Parents of Children with Autism Spectrum Disorder. Aust. New Zealand J. Fam. Ther..

[14] Siller M., Swanson M., Gerber A., Hutman T., Sigman M. (2014). A Parent-Mediated Intervention That Targets Responsive Parental Behaviors Increases Attachment Behaviors in Children with Asd: Results from a Randomized Clinical Trial. J. Autism Dev. Disord..

[15] George C., Judith S. (2008). The Caregiving System: A Behavioral Systems Approach to Parenting. Handb. Attach. Theory Res. Clin. Appl..

[16] Fonagy P., Miriam S., Howard S., George S.M., Anna C.H. (1991). The Capacity for Understanding Mental States: The Reflective Self in Parent and Child and Its Significance for Security of Attachment. Infant Ment. Health J..

[17] Luyten P., Liesbet N., Peter F., Linda C.M. (2017). Parental Reflective Functioning: Theory, Research, and Clinical Applications. Psychoanal. Study Child.

[18] Stern D.N. (2004). The Present Moment in Psychotherapy and Everyday Life (Norton Series on Interpersonal Neurobiology).

[19] Teague S.J., Gray K.M., Tonge B.J., Newman L.K. (2017). Attachment in Children with Autism Spectrum Disorder: A Systematic Review. Res. Autism Spectr. Disord..

[20] Leadbitter K., Macdonald W., Taylor C., Buckle K.L. (2020). Parent Perceptions of Participation in a Parent-Mediated Communication-Focussed Intervention with Their Young Child with Autism Spectrum Disorder. Autism.

[21] Dewey J. (1908). What Does Pragmatism Mean by Practical?. J. Philos. Psychol. Sci. Methods.

[22] Brinkmann S. (2014). Unstructured and Semi-Structured Interviewing. The Oxford Handbook of Qualitative Research.

[23] Ainsworth M.D.S., Mary B., Everett W., Sally W. (1978). Patterns of Attachment.

[24] Sharp C., Peter F. (2008). The Parent’s Capacity to Treat the Child as a Psychological Agent: Constructs, Measures and Implications for Developmental Psychopathology. Soc. Dev..

[25] Van Nes F., Tineke A., Hans J., Dorly D. (2010). Language Differences in Qualitative Research: Is Meaning Lost in Translation?. Eur. J. Ageing.

[26] Thompson J. (2022). A Guide to Abductive Thematic Analysis. Qual. Rep..

[27] Atkinson P., Amanda C., Sara D. (2003). Key Themes in Qualitative Research: Continuities and Changes.

[28] Reichertz J. (2014). Induction, Deduction, Abduction. The SAGE Handbook of Qualitative Data Analysis.

[29] George C., Brandt K., Perry B., Tronick E. (2014). Attachment Theory: Implications for Young Children and Their Parents. Infant and Early Childhood Mental Health: Core Concepts and Clinical Practice.

[30] Ainsworth M.D.S., Silvia M.B., Donelda J.S. (2013). Infant–Mother Attachment and Social Development: ‘Socialisation’ as a Product of Reciprocal Responsiveness to Signals, In Becoming a Person.

[31] Slade A. (2009). Mentalizing the Unmentalizable: Parenting Children on the Spectrum. J. Infant Child Adolesc. Psychother..

[32] Grossmann K.E., Karin G., Franz H., Ulrike W. (1981). German Children’s Behavior Towards Their Mothers at 12 Months and Their Fathers at 18 Months in Ainsworth’s Strange Situation. Int. J. Behav. Dev..

[33] Bretherton I. (2014). Fathers in Attachment Theory and Research: A Review. Emerg. Top. Father Attach..

[34] Tangney J.P., Ronda L.D. (2003). Shame and Guilt.

[35] Winnicott D.W. (1956). Primary Maternal Preoccupation. The Maternal Lineage: Identification, Desire, and Transgenerational Issues.

[36] Trembath D., Gurm M., Scheerer N.E., Trevisan D.A., Paynter J., Bohadana G., Roberts J., Iarocci G. (2019). Systematic Review of Factors That May Influence the Outcomes and Generalizability of Parent-Mediated Interventions for Young Children with Autism Spectrum Disorder. Autism Res..

[37] Cossette-Côté F., Bussières E.L., Dubois-Comtois K. (2022). The Association between Maternal Sensitivity/Availability and Attachment in Children with Autism Spectrum Disorder: A Systematic Review and Meta-Analysis. Curr. Psychol..

[38] De Wolff M.S., Marinus H.V.I. (1997). Sensitivity and Attachment: A Meta-Analysis on Parental Antecedents of Infant Attachment. Child Dev..

[39] Milton D.E.M. (2012). On the Ontological Status of Autism: The ‘Double Empathy Problem’. Disabil. Soc..

[40] Marcinechová D., Lucia Z., Katarína L. (2023). Self-Forgiveness, Guilt, Shame, and Parental Stress among Parents of Children with Autism Spectrum Disorder. Curr. Psychol..

[41] Allgood N. (2005). Parents’ Perceptions of Family-Based Group Music Therapy for Children with Autism Spectrum Disorders. Music Ther. Perspect..

[42] Hodgson A.R., Grahame V., Garland D., Gaultier F., Lecouturier J., Le Couteur A. (2018). Parents’ Opinions About an Intervention to Manage Repetitive Behaviours in Young Children with Autism Spectrum Disorder: A Qualitative Study. J. Appl. Res. Intellect. Disabil..

[43] Pickard K.E., Wainer A.L., Bailey K.M., Ingersoll B.R. (2016). A Mixed-Method Evaluation of the Feasibility and Acceptability of a Telehealth-Based Parent-Mediated Intervention for Children with Autism Spectrum Disorder. Autism.

[44] Stahmer A.C., Lauren B.-F., Sarah R.R., Julia T.S., Joshua D.F., Karyn S., Tiffany W. (2017). Parent Perceptions of an Adapted Evidence-Based Practice for Toddlers with Autism in a Community Setting. Autism.

